# Circulating miRNome profiling in Moyamoya disease-discordant monozygotic twins and endothelial microRNA expression analysis using iPS cell line

**DOI:** 10.1186/s12920-018-0385-3

**Published:** 2018-08-29

**Authors:** Haruto Uchino, Masaki Ito, Ken Kazumata, Yuka Hama, Shuji Hamauchi, Shunsuke Terasaka, Hidenao Sasaki, Kiyohiro Houkin

**Affiliations:** 1 Department of Neurosurgery, Hokkaido University Graduate School of Medicine, North 15 West 7, Sapporo, 0608638 Japan; 2 Department of Neurology, Hokkaido University Graduate School of Medicine, Sapporo, Japan

**Keywords:** Circulating microRNA, Moyamoya disease, Discordant monozygotic twins, iPS cells, Endothelial cells

## Abstract

**Background:**

Moyamoya disease (MMD) is characterized by progressive stenosis of intracranial arteries in the circle of Willis with unknown etiology even after the identification of a Moyamoya susceptible gene, RNF213. Recently, differences in epigenetic regulations have been investigated by a case-control study in MMD. Here, we employed a disease discordant monozygotic twin-based study design to unmask potential confounders.

**Methods:**

Circulating genome-wide microRNA (miRNome) profiling was performed in MMD-discordant monozygotic twins, non-twin-MMD patients, and non-MMD healthy volunteers by microarray followed by qPCRvalidation, using blood samples. Differential plasma-microRNAs were further quantified in endothelial cells differentiated from iPS cell lines (iPSECs) derived from another independent non-twin cohort. Lastly, their target gene expression in the iPSECs was analyzed.

**Results:**

Microarray detected 309 plasma-microRNAs in MMD-discordant monozygotic twins that were also detected in the non-twin cohort. Principal component analysis of the plasma-microRNA expression level demonstrated distinct 2 groups separated by MMD and healthy control in the twin- and non-twin cohorts. Of these, differential upregulations of hsa-miR-6722-3p/− 328-3p were validated in the plasma of MMD (absolute log2 expression fold change (logFC) > 0.26 for the twin cohort; absolute logFC > 0.26, *p* < 0.05, and q < 0.15 for the non-twin cohort). In MMD derived iPSECs, hsa-miR-6722-3p/− 328-3p showed a trend of up-regulation with a 3.0- or higher expression fold change. Bioinformatics analysis revealed that 41 target genes of miR-6722-3p/− 328-3p were significantly down-regulated in MMD derived iPSECs and were involved in STAT3, IGF-1-, and PTEN-signaling, suggesting a potential microRNA-gene expression interaction between circulating plasma and endothelial cells.

**Conclusions:**

Our MMD-discordant monozygotic twin-based study confirmed a novel circulating microRNA signature in MMD as a potential diagnostic biomarker minimally confounded by genetic heterogeneity. The novel circulating microRNA signature can contribute for the future functional microRNA analysis to find new diagnostic and therapeutic target of MMD.

**Electronic supplementary material:**

The online version of this article (10.1186/s12920-018-0385-3) contains supplementary material, which is available to authorized users.

## Background

Moyamoya disease (MMD) is a rare cerebrovascular disorder with unknown cause, characterized by progressive occlusion of the internal carotid arteries and their main branches in the circle of Willis along with the development of an abnormal collateral network known as “moyamoya vessels” [1]. The predominant histopathological feature of MMD is fibrocellular thickening of the intima and attenuation of the media in the affected arterial wall [2–4]. Emerging next generation studies revealed novel genetic aspects of MMD including disease susceptible polymorphisms across the genome in diverse ethnicities [5–7]. In particular, identification of RNF213 gene as a MMD-susceptible gene provided cutting edge impact on MMD research [8, 9]. Endothelial cell (EC) dysfunction has been demonstrated in vitro using iPS cell lines with an RNF213 gene polymorphism (i.e., rs112735431) [10–12], although the primary histopathological features were not reproduced in the intracranial cerebral arteries of RNF213 knock-out mice or in transgenic mice overexpressing RNF213 [11, 13–15].

Recently, several case-control studies have investigated the association between microRNA and MMD upon a non-twin cohort basis [16–18]. MicroRNAs constitute short non-coding RNAs that negatively regulate gene expression at a post-transcriptional level [19]. As they are stable in the sera/plasma and can regulate multiple genes, circulating microRNAs are expected to represent potential diagnostic and prognostic biomarkers of several diseases including stroke [19–21]. Moreover, epigenetic disease studies can particularly benefit from disease-discordant monozygotic twin study design by controlling many potential confounders such as genetic factors, age, and sex [20].

We, therefore, conducted circulating genome-wide microRNA (miRNome) profiling in cohorts of MMD-discordant monozygotic twins, non-twin MMD cases, and healthy controls to unmask potential confounders from a previously reported microRNA signature in MMD [16, 17]. We further studied endothelial microRNA expression using iPS cell lines of another independent non-twin cohort as an in vitro MMD model. Using a published endothelial transcriptome microarray dataset, which has been studied in the exact same cohort, microRNA-gene expression network was analyzed. Understanding the circulating microRNA signature from a discordant monozygotic twin-based study may be invaluable for providing novel insights toward blood biomarker discovery. Furthermore, insights from plasma and endothelial microRNA expression relationships may contribute to provide a potential therapeutic target of MMD.

## Methods

A total of 27 Japanese individuals, including 13 patients with MMD and 14 healthy individuals were included. Two subjects with MMD had participated in previous linkage studies [22, 23]. MMD was diagnosed by magnetic resonance angiography (MRA) or catheter angiography in the Department of Neurosurgery at Hokkaido University Hospital or the referral hospitals between 1987 and 2011 based on the published guideline [24]. Quasi-Moyamoya disease was excluded in this study. We used angiographical stages evaluated according to the MRA stage grading system [25] at the closest timing of blood sampling for analysis. The normal control group consisted of healthy volunteers without any recorded or family history of neurological diseases, including MMD. All normal healthy controls were recruited at the Department of Neurology Hokkaido University Graduate School of Medicine, except for one unaffected MMD-discordant monozygotic twin. In the circulating miRNome microarray study, a pair of MMD-discordant monozygotic twins and a non-twin cohort (nine unrelated subjects with MMD and 10 healthy controls) were included. In the experiment using endothelial cells derived from iPS cell line (iPSECs), another independent non-twin cohort (three patients with MMD and three healthy controls) was recruited, comprising exactly same individuals participated in our previous in vitro iPSEC study [12]. Demographics and clinical features of all study participants were summarized in Table 1.

**Table 1:** Demographics and clinical features of the study participants

	Age (y)		Sex	Disease type	Disease stage	Familial MMD	EC/IC bypass	Comorbidity	Brain lesion	rs112735431 (RNF213)
	at sampling	at onset								
Circulating miRNome microarray analysis										
MMD-Discordant Monozygotic Twins										
Affected Twin	12	3	F	TIA	3	Yes	Yes	No	No	AG
Non-affected Twin	12	–	F	–	–	Yes	–	No	No	AG
Non-Twin cohort										
MMD, n = 9	49.7 ± 5.4	37.3 ± 7.3	66.7%	All TIA	3	56.0%	100%	11%	67%	All AG
Control, *n* = 10	51.7 ± 3.6	–	70%	–	–	0%	0%	0%	–	All GG
*P*-value	0.75		1.0							
iPSEC-microRNA and gene expression analysis										
MMD, n = 3	41.0 ± 3.6	39.1 ± 4.0	67%	All TIA	3	TIA	67%	33%	0%	All AG
Control, n = 3	50.0 ± 2.0	–	0%	–	–	0%	0%	0%	–	All GG

Disease stage indicates MRA grade of the advanced hemisphere if a difference between hemispheres exists. Comorbidity observed in study participants was hypertension. Age (years) was expressed as the mean ± SEM and Disease stage was expressed as median in the non-twin cohort. *F* female, EC/IC bypass: extracranial-intracranial bypass, *TIA* transient ischemic attack. A: minor and G: major allele for rs112735431 of the *RNF213* gene

Detailed methods for blood sample and iPSEC collection/purification, RNA isolation or extraction from plasma or iPSECs, microRNA expression microarray and quantitative real-time polymerase chain reaction (qPCR) analysis, bioinformatics analysis for microRNA-gene expression network and molecular pathway analysis, as well as statistics were provided in supplementary materials and methods (see Additional file 1: Supplementary materials and methods). In brief, differential plasma-microRNA expression microarray analysis was performed in MMD-discordant twin and in non-twin cohorts, respectively. In MMD-discordant monozygotic twins, a set of differential plasma-microRNAs was identified that exhibited a greater than 0.26-absolute expression log fold change between the affected and non-affected monozygotic twins. In the non-twin cohort, a set of differential plasma-microRNAs was identified that exhibited a greater than 0.26-absolute expression log fold change and a less than 0.05 *p*-value computed by unpaired t-test corrected by Welch’s method between MMD and controls followed by multiple testing correction using Storey’s Bootstrapping false discovery rate (FDR) method (q value < 0.15). Raw data of the microarray experiments can be accessed through the National Center for Biotechnology Information Gene Expression Omnibus (NCBI GEO accession number GSE100488).

## Results

### Circulating miRNome profiling demonstrated distinct 2 groups separated by MMD and healthy control

**Fig. 1:**
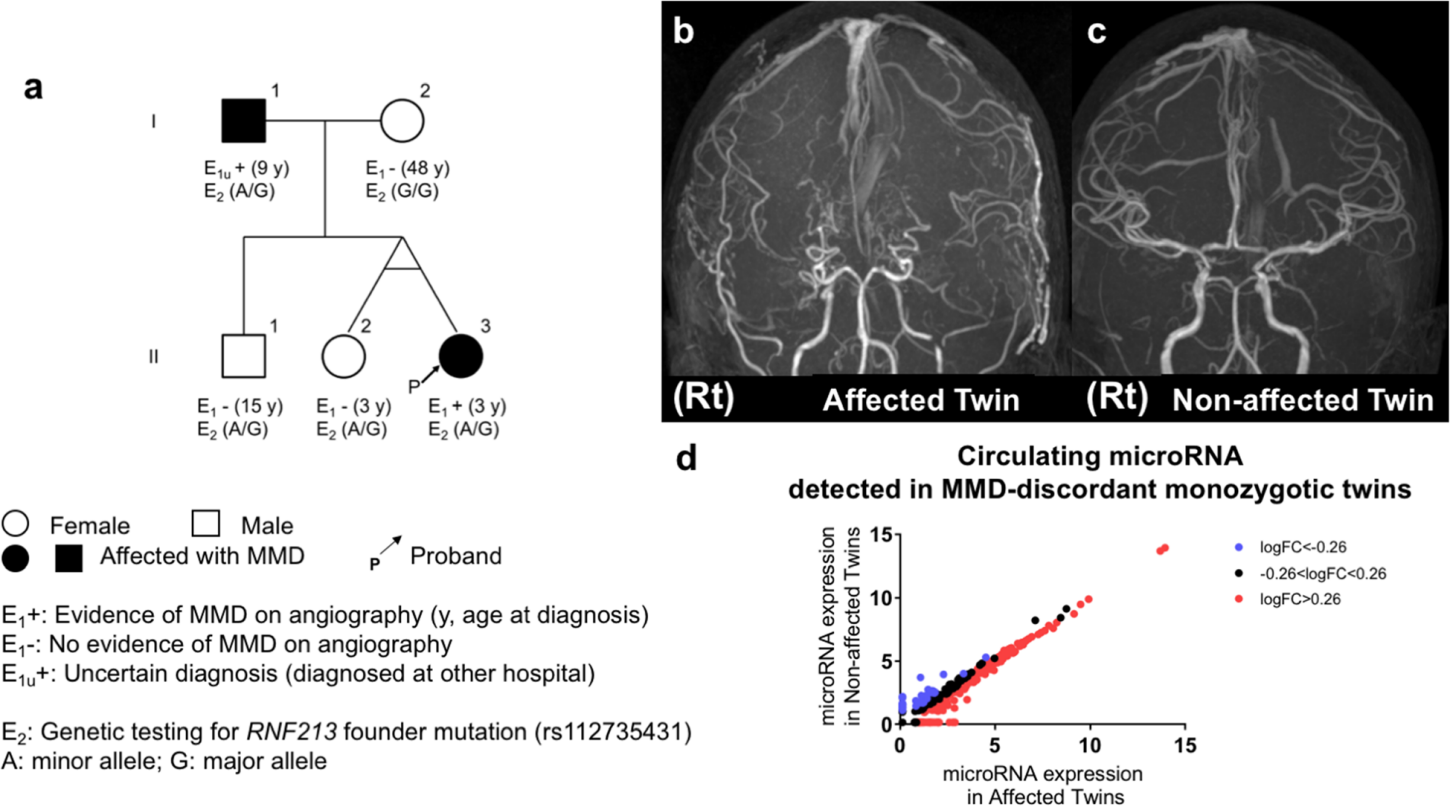
Family pedigree, angiographical features, and plasma-microRNA expression in the Moyamoya disease-discordant monozygotic twins. **a** Family pedigree tree demonstrating the family members of the MMD-discordant monozygotic twins. Individuals I-1 and II-3 had never participated in previous linkage studies. The affected twin (II-3) experienced an epileptic seizure and transient paraparesis when crying at 3 years of age and underwent EC/IC bypass. She did not develop any recurrent stroke, including TIA after bypass surgeries. The non-affected twin (II-2) underwent MRI and MR angiography (MRA) scans when she was 3 and 12 years old without any evidence of cerebrovascular diseases, including MMD. Individual I-1 was diagnosed as MMD when he was 9 years old and underwent bypass surgeries in another hospital. Individuals I-2 and II-1 also provided blood samples and had MRI/MRA scans in our hospital. Their MRI/A showed no evidence of MMD (data not shown). **b**-**c** MRA demonstrates cerebral arteries of the affected twin (II-3) when she was 12 years old (**b**), showing severe stenosis of ICAs and proximal MCAs on both hemispheres and complete disappearance of ACA on the right hemisphere, indicating MRA grade 3 on both hemispheres. Distal portions of bilateral MCAs are depicted by blood supplies from EC/IC bypasses via superficial temporal arteries, whereas MRA of the non-affected twin (II-2) when she was 12 years old demonstrates no steno/occlusive change in the cerebral arteries (**c**). “Rt” indicates the right hemisphere. **d** Scatter plots demonstrate the plasma-microRNA expression for 309 plasma-microRNAs detected in the MMD-discordant monozygotic twins by microarray. Horizontal and vertical axes indicate normalized log2-microRNA expression signals for each microRNA in the affected and non-affected twins, respectively. Red dots indicate microRNAs exhibiting a 0.26- or greater log2-fold change and blue dots indicate microRNAs exhibiting a − 0.26 or less log2-fold change in the affected twin relative to the non-affected twin. Black dots indicate microRNAs exhibiting log2-fold change in between − 0.26 and 0.26

We recruited a pair of MMD-discordant monozygotic twins with familial occurrence of MMD as shown in Fig. 1a to conduct disease-discordant monozygotic twin-based circulating miRNome profiling. Although familial occurrence of MMD comprises approximately 15% of reported cases in Japan, MMD-discordant monozygotic twins are extremely rare [3]. MRAs (Fig. 1b-c) demonstrated bilateral occlusion of ICAs with developed moyamoya vessels in the affected twin (II-3 in the pedigree tree), whereas ICAs of the non-affected twin (II-2) were intact. Genome-wide microRNA expression microarray (SurePrint® G3 Human miRNA microarray (Release 21.0, Agilent)) detected 309 plasma-microRNAs (12.1% of 2549 detectable microRNAs) in this pair of MMD-discordant monozygotic twins. Of these, 151 microRNAs were up-regulated and 36 were down-regulated in the affected compared to the non-affected twin (imposed threshold for expression log2-fold change (logFC); 0.26) (Fig. 1d). The detection rate of plasma-microRNA was higher than that reported in the manufacturer’s application note, and comparable with that in a previous publication studying circulating microRNA in MMD using the same microarray platform [16]. The top five up-regulated plasma-microRNAs in the affected twin were hsa-miR-150-5p (logFC: 2.2), hsa-let-7a-5p (logFC: 2.1), hsa-miR-122-5p (logFC: 2.0), hsa-miR-4419b (logFC: 1.7), and hsa-miR-126-3p (logFC: 1.6), whereas the top five down-regulated plasma-microRNAs were hsa-miR-144-3p (logFC: − 2.1), hsa-miR-8073 (logFC: − 1.5), hsa-miR-6741-5p (logFC: − 1.4), hsa-miR-1237-3p (logFC: − 1.1), and hsa-miR-33b-3p (logFC: − 1.0).

**Fig. 2:**
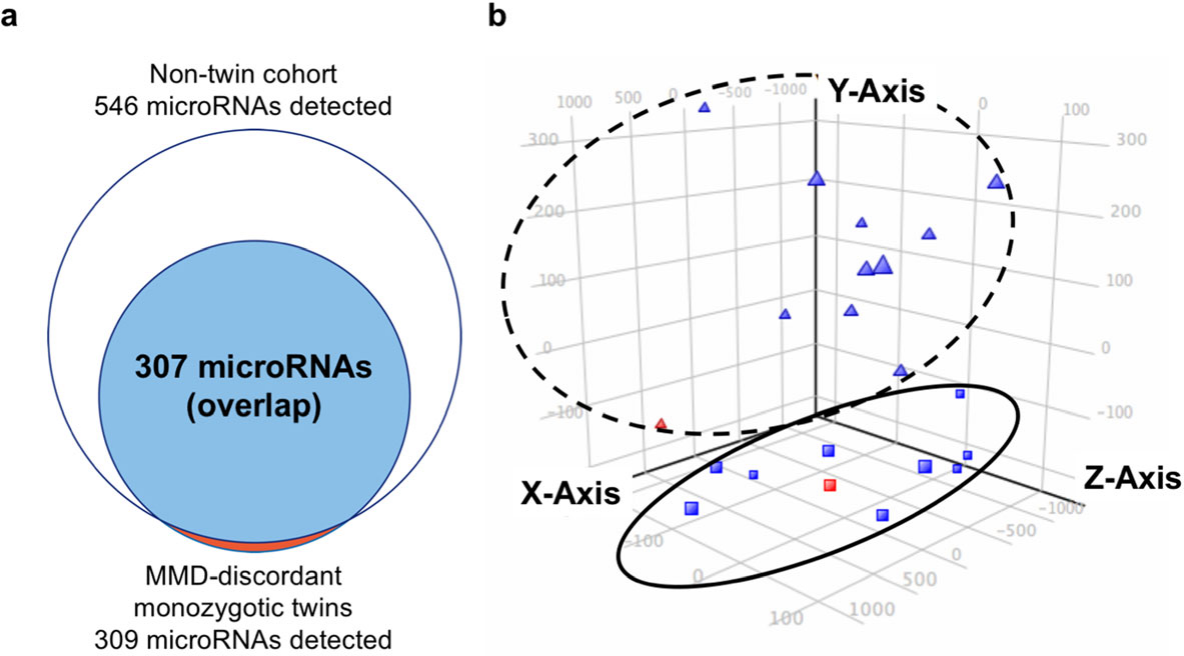
Circulating microRNA expression profiling in the MMD-discordant monozygotic twins and non-twin cohorts. **a** Venn diagram depicts a white circle as a collection of 546 plasma-microRNAs detected in the non-twin cohort (9 MMDs and 10 controls) and a red circle as a collection of 309 plasma-microRNAs detected in the discordant twins. The overlapping closed blue circle represents a set of 307 plasma-microRNAs, indicating predominant plasma-microRNAs detected in the discordant twins (99.4%) were also detected in the non-twin cohort. **b** Three-dimensional PCA plots demonstrate 2 distinct clusters in the circulating microRNA profile separated by MMD and controls. Each dot represents the expression profile of the 309 plasma-microRNAs detected by microarray in the discordant twins without any differential expression analysis. The affected MMD-discordant monozygotic twin (red square) and MMD patients in the non-twin cohort (blue squares) can be grouped (circled in solid line), whereas the non-affected twin (red triangle) and healthy control individuals in the non-twin cohort (blue triangles) can also be grouped (circled in dotted line). Component 2 (Y-axis) was the main component separating MMD and non-MMD control, including MMD-discordant monozygotic twins

Using the same microarray platform, we studied circulating miRNome profiles in nine Japanese patients with MMD and 10 unrelated healthy individuals as a non-twin cohort, identifying 546 plasma-microRNAs with detection call (21.4% of 2549 detectable microRNAs). Because microRNAs detected in the twins were predominantly detected in the non-twin cohort (Fig. 2a), we performed a principal component analysis for the 309 plasma-microRNA level in the discordant twins and the non-twin cohort together (Fig. 2b) and identified distinct 2 clusters. One cluster included the affected monozygotic twin and patients with MMD from the non-twin cohort. The other included non-affected monozygotic twins and healthy controls. Component 2 (Y-axis) was the main component separating MMD and non-MMD control. Top 30 microRNAs with higher absolute PCA scores for each microRNA for the component 2 were listed, showing top 30 plasma microRNAs contributing group segregation between MMD and control (Please see Additional file 2: Table S1). This result suggested that the circulating microRNA expression pattern was associated with MMD even in the MMD-discordant monozygotic twins. Because pediatric monozygotic twins share genetic variants predominantly [20] and many life style environments, differential microRNAs may contribute to the discordance of MMD in the monozygotic twins with fewer confounders relative to a general non-twin based case-control study.

### Differential microRNA expression analysis highlighted 17 plasma-microRNAs in MMD

**Fig. 3:**
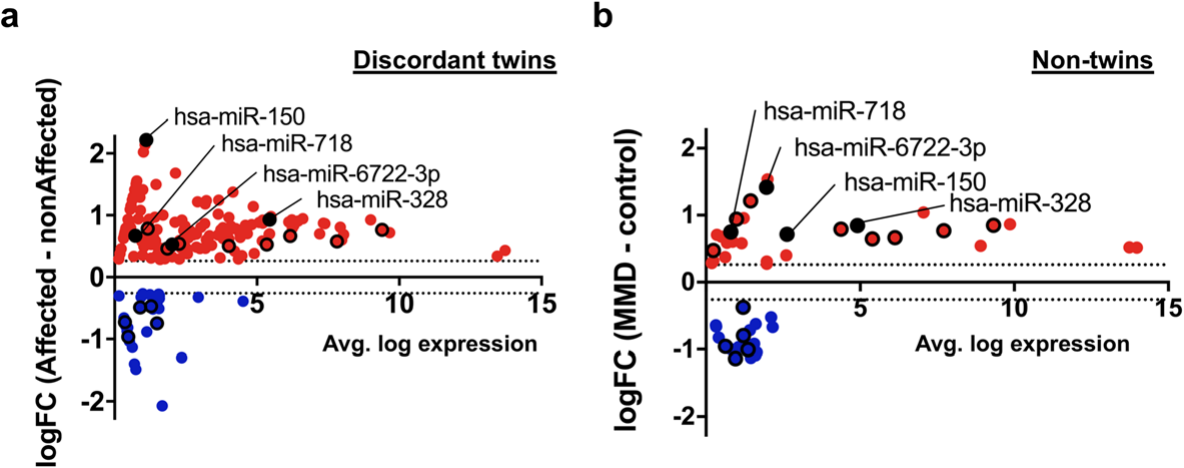
Differential plasma microRNA expression profile in the twin and non-twin cohorts. a-b M-A plot (M = log2 fold change, A = averaged log2 expression) demonstrated the 187-differential plasma-microRNAs (151 up-regulated and 36 down-regulated) in the MMD-discordant monozygotic twins (threshold: absolute log-FC > 0.26; (**a**)), and 49-differential plasma-microRNAs (30 up-regulated and 19 down-regulated) in the non-twin cohort (threshold: absolute log-FC > 0.26 and *p* < 0.05; (**b**)). Seventeen plasma-microRNAs were further highlighted with black circle that showed significant differential expression between MMD and non-MMD both in the twin and non-twin cohort. Of these, 4 microRNAs shown in the plot were validated by real-time qPCR

Based on the distinct circulating microRNA profile associated with MMD, we next performed differential microRNA expression analysis between MMD and healthy controls in the MMD-discordant monozygotic twin and non-twin cohorts using microarray dataset. In the MMD-discordant monozygotic twins, we found 187 differential plasma-microRNAs (151 up-regulated and 36 down-regulated in the affected twin; imposed threshold: absolute logFC > 0.26; Fig. 3a), whereas in the non-twin cohort, we found 49 differential plasma-microRNAs (30 up-regulated and 19 down-regulated in MMD; imposed threshold: absolute logFC > 0.26, *p* < 0.05, q < 0.15; Fig. 3b). From these independent sets of differential plasma-microRNAs, we identified 17 plasma-microRNAs that overlapped between the twin and non-twin cohorts. We confirmed that these plasma-microRNAs were consistently up- (12 microRNAs) or down-regulated (five microRNAs) in the affected monozygotic twin and patients with MMD in the non-twin cohort (Table 2). Of note, 12 microRNAs, including hsa-miR-6722-3p, hsa-miR-328, and hsa-miR-150 were found in the top 30 microRNA list correlated with MMD based on the principal component analysis (Please see Additional file 2: Table S1).

**Table 2:** Differential plasma-microRNAs from discordant monozygotic twin-based study

MicroRNA ID	Discordant twins	Non-twin cohort		
	Log2 fold change	Log2 fold change	p value	q value
hsa-miR-150	2.218	0.714	0.28	0.123
hsa-miR-328	0.931	0.845	0.028	0.123
hsa-miR-3610	0.786	0.475	0.019	0.105
hsa-miR-6089	0.765	0.850	0.005	0.045
hsa-miR-718	0.669	0.749	0.030	0.128
hsa-miR-3665	0.665	0.665	0.023	0.120
hsa-miR-6800-5p	0.576	0.769	0.004	0.042
hsa-miR-762	0.540	1.215	0.009	0.060
hsa-miR-6850-5p	0.528	0.646	0.031	0.128
hsa-miR-6722-3p	0.527	1.415	0.001	0.038
hsa-miR-940	0.503	0.791	0.002	0.038
hsa-miR-4532	0.464	0.940	0.024	0.121
hsa-miR-7845-5p	−0.468	−1.004	0.002	0.038
hsa-miR-623	−0.491	−0.797	0.027	0.123
hsa-miR-595	−0.726	−0.958	0.005	0.045
hsa-miR-6737-3p	−0.746	−0.373	0.000	0.019
hsa-miR-4481	−0.965	−1.136	0.002	0.038

Table listed 17 differential microRNAs that were consistently up- or down-regulated in the affected monozygotic twin and patients with MMD in the non-twin cohort. Log2 fold change indicates relative plasma-microRNA expression level in MMD to control

A total of 8586 messenger RNAs (mRNAs) were predicted as “target genes” of these 17 differential microRNAs by the IPA® tool and miRmap web interface [26]. We further filtered these 8586 genes and obtained 1069 target gene names according to the IPA knowledge-based disease or function terms, including “Cardiovascular diseases”, “Neurological diseases”, and “Vascular physiology” as relevant genes associated with MMD. Table S2 shows the number of target genes for each of the 17 differential microRNAs in detail (see Additional file 3: Table S2).

These 1069-target genes included genes known to be associated with MMD [3, 4, 27], such as RNF213, fibroblast growth factors (FGF2, 7, 14, and 17), hepatocyte growth factor (HGF), platelet derived growth factor B (PDGFB), tumor transforming growth factor beta receptors (TGFBR1 and 2), tumor necrosis factor superfamily member genes (TNFSF4 and 12), and vascular endothelial growth factor A (VEGF A). Notably, hsa-miR-6722-3p can regulate multiple MMD-related genes including RNF213, FGF2 (also known as basic FGF), insulin like growth factor 1 receptor (IGF1R), as well as kinase insert domain receptor (KDR, also known as VEGFR2). Whereas, hsa-328-3p can regulate corticotropin releasing hormone (CRH), protein kinase, cGMP-dependent type I (PRKG1), progesterone receptor (PGR), and others, which are known to be associated with vascular endothelial cell or smooth muscle cell function.

### qPCR verification of hsa-miR-328-3p and − 6722-3p in the plasma and iPSECs

**Fig. 4:**
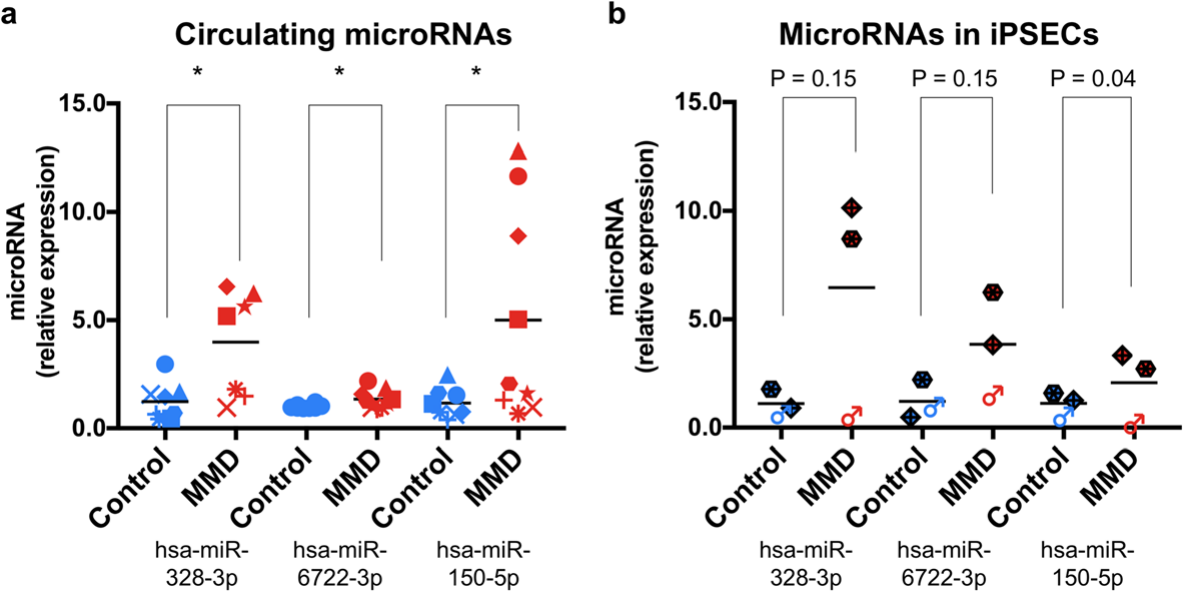
Quantification of microRNA expression in the plasma and iPSECs. **a** Column scatter graphs demonstrating relative plasma-microRNA expression levels for hsa-miR-328-3p (left), − 6722-3p (middle), and 150-5p (right), quantified by real-time PCR in the non-twin cohort (MMD *n* = 9; red dots, control *n* = 8; blue dots). There was a significant difference in each plasma-microRNA expression level between MMD and control groups (hsa-miR-328-3p, 4.0 ± 0.9 and 1.2 ± 0.3, *p* = 0.011; hsa-miR-6722-3p, 1.4 ± 0.1 and 1.0 ± 0.03, *p* = 0.049, hsa-miR-150-5p, 5.0 ± 1.6 and 1.2 ± 0.2, *p* = 0.043, respectively. *p < 0.05). Data represent the mean ± SEM. **b** Column scatter graphs demonstrating relative iPSEC-microRNA expression levels for hsa-miR-328-3p (left), − 6722-3p (middle), and 150-5p (right), quantified by real-time PCR in another non-twin cohort (MMD *n* = 3; red dots, control n = 3; blue dots, please refer to Table 1). There was a trend of difference in the respective hsa-miR-328-3p and − 6722-3p expression levels in iPSECs between MMD and control groups (hsa-miR-328-3p, 6.5 ± 3.0 and 1.1 ± 0.3, *p* = 0.15; hsa-miR-6722-3p, 3.8 ± 1.4 and 1.2 ± 0.5, *p* = 0.15). Conversely, no trend of difference was observed in hsa-miR-150-5p between MMD and control groups (2.1 ± 1.0 and 1.1 ± 0.3, *p* = 0.40, respectively). Data represents as mean ± SEM. Each individual data was plotted by same color and same shape code

To validate the results from microarray analysis, we performed real-time qPCR for the plasma-microRNAs of interest using a modified ΔΔCT method [28]. Thus, from the 17 differential microRNAs highlighted by the plasma-microRNA microarray analysis (Table 2), we quantified the relative expression level of hsa-miR-150-5p, − 328-3p, − 6722-3p, and − 718 using plasma samples of the non-twin cohort because these microRNAs were more up-regulated in the affected MMD-discordant twins, more significantly up-regulated in patients with MMD in the non-twin cohort, or have target genes relevantly related to MMD (see the rationale for this selection in Additional file 1: Supplementary materials and methods). We confirmed that hsa-miR-150-5p, − 328-3p, and − 6722-3p were significantly up-regulated in the plasma of MMD (*p* < 0.05) (Fig. 4a), which was consistent with the microarray result. Conversely, hsa-miR-718 was not amplified by real-time qPCR presumably due to low abundance in the plasma samples analyzed in this study, which was suggested by lower expression signal in the microarray result (Fig. 3b). Although Fig. 4a indicated that there were 2 types of the miR-328-3p and 150-5p plasma expression pattern (elevated and non-elevated), these 4 elevated samples did not match each other. There was no common trend in these elevated samples.

To investigate the expression levels of these microRNAs in the ECs of MMD, we employed an in vitro MMD-iPSEC model, comprising differentiated and cultured ECs from iPS cell lines derived from another independent non-twin cohort (Table 1). The purity of iPSECs were confirmed by FACS plot (Please see Additional file 4: Figure S1). We found a trend of up-regulation of hsa-miR-328-3p and − 6722-3p in the iPSECs derived from MMD with a 3.0- or higher expression fold change, although it did not reach statistical significance by t-test due to small sample size. Whereas, there was no trend of difference in hsa-miR-150-5p expression level in iPSECs between MMD and the control (*p* = 0.40) (Fig. 4b).

### MicroRNA-gene expression network analysis suggests a regulatory role of hsa-miR-328-3p/− 6722-3p involving biological pathways in endothelial cells of MMD

Based on the results from qPCR validations, hsa-miR-328-3p and − 6722-3p were identified as differential microRNAs of MMD, which may be associated with the phenotypic discordance in the MMD-discordant monozygotic twins. As these microRNAs showed a significant upregulation in the plasma in MMD and a trend of differential up-regulation in the MMD-iPSECs, we interrogated the regulatory role of hsa-miR-328-3p/− 6722-3p in their target gene expression. Based on the aforementioned target gene prediction, 327 target genes of hsa-miR-328-3p/− 6722-3p were identified (287 for hsa-miR-6722-3p and 48 for hsa-miR-328-3p) (please see Additional file 3: Table S2). We analyzed an experimentally validated microarray dataset from our previous publication that investigated gene expression profile in the iPSECs derived from the same MMD and healthy control individuals (*n* = 3, respectively, Table 1) as well as differentiated/cultured under identical conditions [12]. Of these 327 target genes, 41 genes (12.5%) were significantly down-regulated (logFC range − 2.0 to − 0.6, *p* < 0.05, q < 0.15), and 14 genes (4.3%) were significantly up-regulated (log-FC: range 0.6 to 2.6, p < 0.05, q < 0.15) in MMD-iPSECs. The gene list was presented in the Additional file 5: Table S3. This result suggested that significant down-regulation of these 41 genes might be associated with epigenetic regulation of the up-regulated hsa-miR-328-3p and − 6722-3p in MMD-iPSECs.

**Fig. 5:**
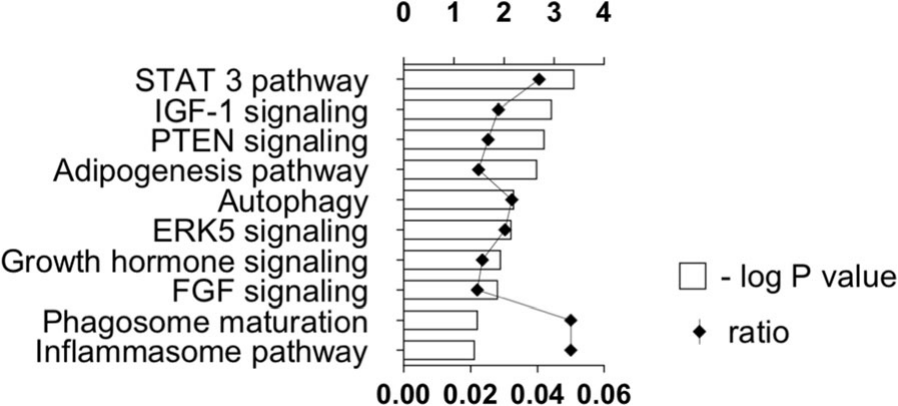
Top 10 canonical pathways involved by the 41 significantly down-regulated genes in MMD-iPSECs. Graph showing the top 10 canonical pathways from IPA® pathway analysis involved by the 41 significantly down-regulated genes in MMD-iPSECs that were predicted as target genes of significantly up-regulated hsa-miR-328-3p and − 6722-3p in MMD-iPSECs. The ratio expresses the number of differentially down-regulated genes in our dataset over the total number of genes in each canonical pathway based on the IPA knowledge database (represented in the lower X axis). *P* values for the significance of each pathway were computed based on the number of genes involved in the pathway by using Fisher’s exact test, and negatively log10 transformed (represented in the upper X axis)

We annotated these 41 down-regulated genes in the MMD-iPSECs using IPA tool. Thus, the least five genes down-regulated in the MMD-iPSECs were ADAM metallopeptidase domain 12 (ADAM12, log-FC: − 2.0), cyclin dependent kinase 6 (CDK6, log-FC: − 2.0), additional sex combs like 3, transcriptional regulator (ASXL3, log-FC: − 1.9), pappalysin 1 (PAPPA, also known as insulin-like growth factor-dependent IGF binding protein-4 protease, log-FC: − 1.8), and RNF213 (log-FC: − 1.6). IPA canonical pathway analysis for these 41 down-regulated genes demonstrated the top three canonical pathways, namely the signal transducer and activator of transcription 3 (STAT3) pathway, insulin-like growth factor 1 (IGF-1) signaling, and phosphatase and tensin homolog (PTEN) signaling, which are known angiogenesis and vascular homeostasis-related pathways (Fig. 5). In addition, these genes were also involved in autophagy, extracellular signal regulated kinase 5 (ERK5) signaling, phagosome maturation, and the inflammasome pathway, which comprise inflammation or infection-related signaling pathways.

## Discussion

We conducted circulating genome-wide microRNA profiling in MMD using a disease-discordant monozygotic twins-based design to discover a less confounded microRNA signature. Result was validated in an independent non-twin MMD cohort. Thus, our strategy could reduce the effect of many possible confounders (i.e., genetic heterogeneity) affecting the findings from a general non-twin-based case-control study. We observed that plasma-microRNA expression profiling could clearly separate MMD and healthy control in the MMD-discordant monozygotic twin- and the non-twin cohort. From differential microRNA expression analysis between MMD and controls, a novel 17 differential plasma-microRNAs signature was identified, since 16 of these plasma-microRNAs have not been reported in the previous exploratory study for circulating microRNA signature in MMD using non-twin based study design [16, 17]. We have successfully validated differential upregulation of hsa-miR-6722-3p/− 328-3p/− 150-5p in the plasma of MMD patients. We also found a trend of upregulation of hsa-miR-6722-3p/− 328-3p in MMD-iPSECs. Furthermore, some of their predicted target genes were significantly downregulated in MMD-iPSECs, suggesting a role of epigenetic regulation of circulating microRNA in endothelial cells in MMD. This is the first observation of possible microRNA-gene expression network between the plasma and endothelial cells in MMD.

### Circulating microRNA signature in MMD in comparison with that from a previous non-twin-based case-control study

Dai et al. first reported 94 differential circulating microRNAs in Chinese Han MMD compared to ethnicity-matched normal controls [16] using the same microarray platform. Zhao et al. next specifically analyzed serum microRNA let-7 family expression in the sera of MMD as a regulator of the RNF213 gene [17]. Because both studies employed a general non-twin-based study design and found circulating microRNA signatures as candidates for blood biomarkers in MMD, we considered that our MMD-discordant monozygotic twin-based design could provide additional insights to their findings. In particular, the MMD-discordant monozygotic twins that participated in the present study shared age, sex, ethnicity, and an RNF213 founder mutation polymorphism (rs112735431). Moreover, the affected twin was clinically stable without recurrent transient ischemic attack (TIA), recent surgery (approximately 9 years prior to blood sampling), or ischemic parenchymal lesion. Therefore, the distinct circulating microRNA profile detected in the discordant twins could likely explain the phenotypic discordance between MMD and non-MMD with minimal confounders. Notably, our group and Dai et al. both demonstrated that hsa-miR-595 constituted a differentially down-regulated plasma-microRNA in MMD [16]. Future study to analyze the functional role of hsa-miR-595 in MMD with different subsets of Asian ethnicity may provide important insights to understand the epigenetic aspects of MMD. However, we also identified numerous discrepancies in the results of circulating microRNA signature in MMD between the two publications. Several possible reasons for this discrepancy exist in addition to the difference in study design. First, the plasma sample processing for the array experiment fundamentally differed between studies. Dai et al. pooled plasma samples from 10 individuals with MMD and controls and undertook two microarray experiments for the two plasma pools, whereas we performed microarray experiments using 21 individual (non-pooled) samples. This difference might affect the cluster analysis used to detect the difference of microRNA profile between MMD and control. Second, the imposed threshold to determine differential microRNA in MMD is different. Third, the subgroup of ethnicity in study participants was also different (Japanese or Chinese-Han). As some differences have been observed in the genetic and epidemiological features of MMD in Chinese Han and Japanese or Korean patients [29], this may result in a difference in the circulating microRNA signature. Future work expanding the study population toward non-Asian MMD would also be invaluable to address the inter-ethnic difference/variability of clinical MMD phenotype [30].

### Plasma and iPSEC microRNA expression and gene regulation in MMD

The differential up-regulation of hsa-mir-6722-3p and − 328-3p in the plasma of MMD was validated in this study. As well, there was a trend of elevated expression of those microRNAs in the iPSECs (3.0- or higher fold change in MMD compared to healthy control), suggesting possible association in the microRNA expression between circulating plasma and endothelial cells in MMD. Although the functional role of circulating microRNAs is often unclear, they are considered to be secreted actively from cells and involved in inter-cellular communications [31]. From the target gene analysis, a set of 41 genes was confirmed as targets of the up-regulated hsa-mir-6722-3p and − 328-3p in MMD-iPSECs and these genes were further experimentally demonstrated to exhibit significant down-regulation in MMD-iPSECs from the same host with exactly the same differentiation/cultural conditions from our previous publication [12]. The molecular types of the 41 gene products include enzyme (i.e., RNF213), growth factor (i.e., FGF), ion channel, kinase, peptidase, phosphatase, transcription regulator (i.e., STAT3), transmembrane receptor (i.e., IGF1R), and transporter (see Additional file 5: Table S3). IPA canonical pathway analysis revealed that these 41 down-regulated genes were involved in angiogenesis, vascular homeostasis-related signaling/pathways (STAT-3 pathway, IGF-1 signaling, and PTEN signaling), or inflammatory-related biological pathways (ERK signaling, inflammasome pathway). These facts suggest a possible plasma microRNA-endothelial gene expression network as an epigenetic regulation of endothelial biological function of MMD. For example, endothelial STAT3 plays a key role in angiogenesis and extracellular matrix remodeling [32]. IGF-1 has been shown to protect vascular function by promoting nitric oxide production from the endothelium and vascular smooth muscle cells [33]. PTEN plays critical roles for vascular homeostasis by regulating signals via multiple vascular growth factors and responses to shear stress via integrins [34, 35]. Taken together, these 41 down-regulated genes in MMD-iPSECs may play a role in endothelial biological functions via the microRNA-gene expression network in MMD. In addition, these 41 genes were also suggested to be involved in other inflammatory-related pathways. A recent study reported that pro-inflammatory cytokines activated the transcription of RNF213, the product of which plays a role in ECs for proper gene expression in response to inflammatory signals from environments [36]. Our data thus provide additional insights into the potential network of RNF213 gene expression regulation via up-regulated hsa-miR-6722-3p/− 328-3p in MMD.

There are several limitations in this study. First, the sample size was small and limited to the Japanese population. Second, this cross-sectional study sampled plasma at a single time point after development of MMD. Third, the disease type of MMD was limited to TIA-type MMD. Fourth, the differentially expressed plasma-microRNAs in MMD that have not been validated by qPCR should be quantified to verify the microarray result, since we have set less stringent cutoff calling to determine differential plasma-microRNAs in the microarray experiment. Finally, functional validation of the differential plasma-microRNAs was lacking not only in the iPSECs but in other cell types, including vascular smooth muscle cells and circulating immune cells (i.e., monocytes, T lymphocytes, and vascular progenitor cells). Addressing these limitations would be invaluable to consolidate the circulating microRNA signature as a blood biomarker in MMD and potentially uncover disease etiology. Although there are many challenges in the clinical trial designs for rare diseases [37], we think it is necessary to conduct a validation study of the present study with the adequate sample size calculation in an independent cohort.

## Conclusions

This study demonstrated a distinct circulating microRNA expression profile in MMD through a unique MMD-discordant monozygotic twin-based study design. A plasma-microRNA dataset presented in this study can provide a seed to discover blood biomarker for MMD with minimal possible confounders. Furthermore, to our knowledge, this is the first study showing that the microRNA-gene expression network may play a role in epigenetic regulation of endothelial function via angiogenesis, vascular homeostasis, and inflammatory-related pathways as a potential therapeutic target for MMD.

## Additional files

Additional file 1: **Supplementary Materials and Methods**. Details supporting the main methods in the manuscript body are provided necessary to comprehend the results. (DOCX 35 kb)

Additional file 2: **Table S1.** A list of microRNAs with respective top 15 and least 15 PCA scores. A list of microRNAs with respective top 15 and least 15 PCA scores (correlation value) for each microRNA for the component 2 (Y-axis) were provided, which separated MMD and control, based on the principal component analysis for the 309 plasma-microRNAs (Please see Fig. 2b). (DOCX 14 kb)

Additional file 3: **Table S2.** Number of filtered target genes and target gene symbols for each of 17 differential circulating microRNAs in MMD, predicted by microRNA Target Filter implemented in IPA® (QIAGEN) and the miRmap web interface. The target genes were filtered based on their biological function analyzed using “Bioprofiler” implemented in IPA® (QIAGEN). As there is some overlap across microRNAs with respect to their target genes, so the total number of target genes at the bottom of the list is not a simple summation of each number of target genes. (DOCX 17 kb)

Additional file 4: **Figure S1.** FACS plot to confirm the purity of iPSECs. The purity of the iPSECs after 5–6 times passages was confirmed as high as 91–97% in all clones using anti-CD31 antibody with the FACS. (TIF 1011 kb)

Additional file 5: **Table S3.** The 41 target genes of hsa-miR-6722-3p/328-3p that were significantly down-regulated in MMD-iPSECs. Expression log2 fold change (log2 FC), *p*-value, and q-value were obtained from our previous publication (Hamauchi et al. 2016 [12]). Molecule type for each gene was obtained from the IPA tool. (DOCX 19 kb)

## Abbreviations

CRH: corticotropin releasing hormone
FGF: fibroblast growth factor
HGF: hepatocyte growth factor
IGF1R: insulin like growth factor 1 receptor
iPSECs: endothelial cells differentiated from iPS cell line
KDR: kinase insert domain receptor
logFC: log2-fold change
miRNome: Circulating genome-wide microRNA
MMD: Moyamoya diease
MRA: magnetic resonance angiography
mRNA: messenger RNA
PDGFB: platelet derived growth factor beta
qPCR: quantitative polymerase chain reaction
TGFBR: tumor transforming growth factor beta receptor
TNFSF: tumor necrosis factor superfamily member genes
VEGFA: vascular endothelial growth factor A
PRKG1: protein kinase, cGMP-dependent type I
PGR: progesterone receptor
ADAM12: ADAM metallopeptidase domain 12
CDK6: cyclin dependent kinase 6
ASXL3: additional sex combs like 3 transcriptional regulator
PAPPA: pappalysin 1
STAT3: signal transducer and activator of transcription 3
IGF-1: insulin-like growth factor 1
PTEN: phosphatase and tensin homolog
ERK5: extracellular signal regulated kinase 5
TIA: transient ischemic attack
