# Supplementary material for: Circulating miRNome profiling in Moyamoya disease-discordant monozygotic twins and endothelial microRNA expression analysis using iPS cell line
Source: BMC Med Genomics. 2018 Aug 29;11:72. doi: 10.1186/s12920-018-0385-3 (PMC6114494; doi:10.1186/s12920-018-0385-3)
Supplement: Supplementary file 1 — Supplementary Materials and Methods. Details supporting the main methods in the manuscript body are provided necessary to comprehend the results. (DOCX 35 kb) [file 12920_2018_385_MOESM1_ESM.docx]

**Supplementary Materials and Methods**

**Blood sample preparation and RNA isolation from plasma**

Whole blood samples were collected in Hokkaido University hospital. In MMD cases, the mean duration between the blood sampling and the latest transient ischemic attack (TIA) or extracranial/intracranial (EC/IC) bypass surgery was 10.3 ± 2.6 or 10.2 ± 2.7 years, respectively. Blood samples were collected into disodium ethylenediaminotetraacetate and PAXgene Blood DNA tubes, centrifuged immediately, and plasma and blood cell pellets were stored at −80 °C until further analysis. Genetic and epigenetic testing was conducted at the Departments of Neurosurgery and Neurology in Hokkaido University, including *RNF213* founder mutation (rs112735431) screening. For the microRNA microarray experiment, total RNA was isolated from 200 uL of the pooled blood plasma using a miRNeasy^®^ Serum/Plasma Kit (QIAGEN Inc., Hilden, Germany) and stored at −80 °C until use. In comparison, for the quantitative real-time PCR (qPCR) experiments, total RNA was isolated from 200 uL of the pooled plasma with the cel-miR-39 Spike-In Kit (NORGEN BIOTEK, Thorold, ON, Canada), using a miRNeasy^®^ Serum/Plasma Kit (QIAGEN) and stored at −80 °C until use. RNA quality was confirmed by electropherogram using the RNA 6000 Pico Kit^®^ for total RNA (Agilent, Santa Clara, CA, USA) and the Agilent 2100 Bioanalyzer^®^. As the RNA integrity number (RIN) provided by the Bioanalyzer takes the entire electrophoretic trace into account, whereas plasma samples contain short fragments of RNA (< 1000 nt) that would be perceived as degraded RNA, we therefore did not interpret low RIN values as degraded RNA in this study.

**RNF213 founder mutation screening**

Genomic DNA was extracted from the white blood cells to identify *RNF213* founder mutation (rs112735431) for all participants in the study by direct sequencing. Sequence reactions and sequence analyses were conducted using a Big Dye Terminator cycle sequencing kit (Thermo Fisher Scientific Inc., Waltham, MA, USA) and an ABI PRISM 3130-Avant Genetic Analyzer (Thermo Fisher Scientific) in Hokkaido University School of Medicine. The primer sequences for the detection of rs11273543 (*RNF213* c.14576G>A (p.R4859K) located in exon 61 based on National Center for Biotechnology Information reference sequence number 1. NM_020914.4.) were as follows: forward; 5’- CTG CAT CAC AGG AAA TGA CAC TG -3’, reverse; 3’- TGA CGA GAA GAG CTT TCA GAC GA -5’. Family members of the monozygotic twins (**Fig. 1a**, Individuals I-1, 2, and II-1) agreed to provide the screening results under written consent, which were conducted for another genetic analysis study (submitted).

**Plasma-microRNA expression microarray and analysis**

Total RNA samples isolated from plasma were processed with the miRNA Complete Labeling and Hyb Kit and microRNA Spike-In Kit (Agilent) according to the manufacturer’s protocol, then hybridized to SurePrint^®^ G3 Human miRNA microarray (Release 21.0, Agilent), which provides a comprehensive coverage of 2549 human microRNA targets. After washing steps, the microarray was scanned using an Agilent SureScan^®^ Microarray Scanner (G2600D) and the raw signal values were obtained and summarized by Feature Extraction^®^ software (version 11.0.1.1, Agilent). All the quality metrics of microarray experiments showed as excellent or good. Using GeneSpring^®^ software (GeneSpring 12.6.1, Agilent), we analyzed the raw data for normalization, cluster analysis, and differential expression analysis. In the first step, the feature extracted text files with raw signal values for each microRNA entity were loaded into GeneSpring^®^. Normalization and log2 transformation of the expression signal values were performed among the 309 microRNAs that were detected in the MMD-discordant monozygotic twins. Thus, the raw signal values of the 309 microRNAs in all 21 samples (a pair of MMD-discordant monozygotic twins and non-twin cohort with 9 MMDs and 10 healthy controls) were normalized by quantile shift. Expression signals after quantile normalization and log2 transformation were summarized as “normalized signals” along with “Systematic Name of microRNA” based on miRbase ver.21.0.

Principal component analysis was performed to display the variability of microRNA expression profiles across samples for the 309 microRNAs detected in the MMD-discordant monozygotic twins. Next, differential plasma-microRNA expression analysis was performed in MMD-discordant twin and in non-twin cohorts, respectively. In MMD-discordant monozygotic twins, a set of differential plasma-microRNAs was identified that exhibited a greater than 0.26-absolute expression log fold change between the affected and non-affected MZ twins. In the non-twin cohort, a set of differential plasma-microRNAs was identified that exhibited a greater than 0.26-absolute expression log fold change and a less than 0.05 p-value computed by unpaired t-test corrected by Welch’s method between MMD cases and controls followed by multiple testing correction using Storey’s Bootstrapping false discovery rate (FDR) method (q value < 0.15). We used a Venn diagram to find consistently elevated or reduced plasma-microRNAs in MMD from the discordant twin and non-twin cohorts.

**iPSEC preparation and RNA extraction**

Endothelial cells (ECs) differentiated from a pooled iPS cell line (iPSEC) were prepared as described in our previous publication (Hamauchi et al. 2016; cited in the main text [12]). In brief, we used pooled, previously established iPS cell lines. These cells had been obtained from a non-twin cohort (3 patients with MMD and 3 controls, listed in the **Table 1** of the main text). Once iPSECs were obtained, purification was performed by fluorescence activated cell sorting (FACS) and cell culture. In brief, the iPS cells at subconfluency were detached using CTK solution consisting of 0.1 mg/mL collagenase IV (Invitrogen), 0.25% trypsin (Invitrogen, Waltham, MA, USA), 0.1 mM CaCl_2_ (Nacalai Tesque, Kyoto Japan) and 20% KSR and seeded onto Matrigel-coated dishes at a ratio of 1:5 to 1:10. iPS cells were treated with 50 ng/mL bone morphogenetic protein 4 (BMP4; R & D Systems, Minneapolis, MN, USA) and 50 ng/mL basic fibroblast growth factor (bFGF; Wako, Osaka, Japan) for the first 24 h. Then, cells were treated with 40 ng/mL vascular endothelial growth factor (VEGF; Invitrogen) and 50 ng/mL bFGF for 2 days; and 40 ng/mL VEGF, 50 ng/mL bFGF, and 20 μmol/L SB431542 (Miltenyi Biotec, Teterow, Germany) for the following 3–4 days. Subsequently, iPSECs were purified using a FITC-conjugated anti-CD31 antibody (1 μg/1 × 10^6^ cells, WM59; BioLegend, San Diego, CA, USA) and APC-conjugated anti-CD144 antibody (0.5 μg/1.0 × 10^6^cells; 16B1, eBioscience, San Diego, CA, USA) with a FACS Aria III (BD Biosciences, San Jose, CA, USA). The purified iPSECs were seeded and maintained on collagen I-coated dishes (CORNING, Armonk, NY, USA) with HuMedia-EB2 medium (KURABO, Osaka, Japan), supplemented with 20 ng/mL VEGF, 25 ng/mL bFGF, 0.5% penicillin and streptomycin (Nacalai Tesque), and 10% fetal bovine serum (Thermo Fisher Scientific). The iPSECs were passaged 5 or 6 times for subsequent experiments. Before RNA extractions for subsequent experiments, the purity of these iPSECs were confirmed as high as 91-97% in all clones using anti-CD31 antibody with the FACS (Please see Additional file 5: Figure S1).

Total RNA was extracted from the lysate of 1.0 × 10^5^ iPSECs using the miRNeasy Mini Kit (QIAGEN) with the cel-miR-39 Spike-In Kit and stored at −80 °C until use. RNA quality was confirmed using the RNA 6000 Pico Kit^®^ for total RNA (Agilent) and the Agilent 2100 Bioanalyzer^®^.

**Quantitative real-time PCR for plasma/iPSEC-microRNA**

To validate the microarray experiments for plasma-microRNAs, we performed qPCR for selected plasma-microRNAs of interest using total RNAs isolated from the plasma of the non-twin cohort (9 MMD and 10 controls). From the final set of differential plasma-microRNAs, we selected hsa-miR-718, -miR-6722-3p, -328-3p, and -150-5p for qPCR validation, based on the following reasons: 1) higher absolute expression log fold change in the affected monozygotic twin, 2) higher significance level (smaller p-value) in the non-twin cohort, or 3) the relevant target gene function of the microRNA, which could be associated with MMD pathophysiology (target gene analysis for microRNAs is described below; “Bioinformatics analysis for microRNA-gene expression network/molecular pathway”). We then performed qPCR using RNAs extracted from iPSECs for the validated plasma-microRNAs to investigate their expression levels in ECs.

The qPCR was performed using TaqMan^®^ microRNA assays (Applied Biosystems, Foster City, CA, USA) with an external spike-in microRNA (cel-miR-39 Spike-In Kit, NORGEN BIOTEK) as a reference control because the Agilent RNA 6000 Pico Kit for total RNA does not provide quantitation accuracy (referencing the manufacturer). Thus, we could not prepare exactly the same amount of total RNA as a starting material for the following reverse transcription (RT) reactions. Therefore, we employed an external spike-in microRNA (cel-miR-39 Spike-In Kit, NORGEN BIOTEK) as a reference control for quantification. For this purpose, we added 33 fmol of cel-miR-39 to each plasma sample or lysate of the iPSECs at the beginning of total RNA isolation or extraction, respectively. Then, first-strand cDNA synthesis was performed according to the manufacturer’s protocol for creating custom RT and pre-amplification (pre-Amp) pools using TaqMan^®^ microRNA assays (Applied Biosystems). Thus, 3 µL of total RNA was processed for RT reaction using the TaqMan^®^ microRNA RT Kit and pooled TaqMan^®^ microRNA Assay primers (Applied Biosystems). The cDNA was subjected to one round of pre-Amp using the TaqMan^®^ PreAmp Master Mix with pooled PreAmp primers (Applied Biosystems). StepOnePlus^®^ real-time PCR system (Applied Biosystems) was used for qPCR. qPCR reaction mixtures were prepared with duplicates using TaqMan^®^ Universal Master Mix II (No AmpErase^®^ UNG (2x)) and TaqMan^®^ MicroRNA Assays (Applied Biosystems), targeting hsa-miR-718 (241118_mat), -miR-6722-3p (474380_mat), -miR-328-3p (000543), and –miR-150-5p (000473) as microRNAs of interest, and cel-miR-39 (464312_mat) as a reference control. qPCR data were analyzed by the modified ΔΔCT method for the correction of primer efficiency as described elsewhere (Pfaffl et al. 2001, cited in the main text[29]). Thus, the relative quantification of a target microRNA in MMD in comparison with a reference microRNA (cel-miR-39) and the healthy control group was performed for every sample. The mathematical model for data analysis was established to calculate the expression level of the target microRNA in MMD relative to normal controls and a reference microRNA (cel-miR-39) based on each primer efficiency (E) for PCR amplification as follows:

Relative expression = (E_target miRNA_)^ΔCt-target (control − MMD)^/ (E_cel-miR-39_)^ΔCt-cel-miR-39 (control − MMD)^

**Bioinformatics analysis for microRNA-gene expression networks and molecular pathways**

Using the Ingenuity Pathway Analysis (IPA^®^) tool (QIAGEN) and the miRmap web interface, the target messenger RNA (mRNA) of microRNA was analyzed to study the microRNA-gene expression network. First, we analyzed the target mRNAs of the 17 differential plasma-microRNAs obtained from microarray analysis using the microRNA Target Filter (content version 33559992, date 2017-03-28) implemented in IPA^®^ (QIAGEN) with a confidence level setting at highly predicted or experimentally confirmed. miRmap web interface was also used with a minimum threshold of miRmap score 90. The target mRNA list was obtained for each microRNA, then we further filtered the target mRNAs based on their biological function using “Bioprofiler” implemented in IPA^®^ (QIAGEN) with a species evidence setting as human only, disease or function filter terms of “vascular physiology” or “neurological diseases” or “cardiovascular diseases”, as well as with an evidence setting at causal relationship only, which allow a focus on relevant target genes associated with MMD.

For iPSEC gene expression microarray data analysis, we obtained the iPSEC gene expression microarray data from our previous publication (Hamauchi et al. 2016, citation in the main text [12]), and raw signal values were uploaded into GeneSpring^®^ 12.6.1 software (Agilent). After quantile normalization within all 6 sample datasets for probes with detection flag calls, log2-fold change of gene expression, p-values, as well as FDR q-values for multiple testing correction were computed by comparing MMD cases and healthy controls (n = 3, respectively). P-values were computed by a Moderate t-test and q-values were computed by Storey’s Bootstrapping method for FDR. Differentially expressed genes (DEGs) were determined by imposing minimum threshold of difference between MMD and controls as absolute log2 fold change > 0.58, p < 0.05, and q < 0.15. Using this dataset, we analyzed microRNA-gene expression networks in the iPSECs for the differential microRNAs identified in the qPCR validation. Thus, the expression levels of the target genes of the differential iPSEC-microRNAs were investigated. The microRNA expression and gene expression (mRNA level) in the iPSECs were analyzed using the *in vitro* MMD iPSEC model from the same cohort.

To study molecular pathways involving genes with microRNA-gene expression interaction in iPSECs of MMD, the gene list and expression fold change data were uploaded into IPA^®^ for the canonical pathway analysis.

**Statistical analysis**

Graphical and statistical analyses were conducted using GraphPad Prism^®^ 7.03 (GraphPad Software, Inc. La Jolla, CA, USA). Continuous values were expressed as the means ± standard error of mean (sem), and discrete variables were expressed as the median. A χ2 test or Fisher’s exact probability test was applied to compare the dichotomous variables between two groups. Continuous variables were compared using the two-tailed unpaired-t test or Mann-Whitney U test between two groups. The microRNA microarray analysis and transcriptome gene expression microarray analysis were performed using GeneSpring^®^ software (GeneSpring 12.6.1, Agilent). P < 0.05 was considered statistically significant.
