# Supplementary material for: Circulating miRNome profiling in Moyamoya disease-discordant monozygotic twins and endothelial microRNA expression analysis using iPS cell line
Source: BMC Med Genomics. 2018 Aug 29;11:72. doi: 10.1186/s12920-018-0385-3 (PMC6114494; doi:10.1186/s12920-018-0385-3)
Supplement: Supplementary file 2 — Table S1. A list of microRNAs with respective top 15 and least 15 PCA scores. A list of microRNAs with respective top 15 and least 15 PCA scores (correlation value) for each microRNA for the component 2 (Y-axis) were provided, which separated MMD and control, based on the principal component analysis for the 309 plasma-microRNAs (Please see Fig. 2b). (DOCX 14 kb) [file 12920_2018_385_MOESM2_ESM.docx]

**Table S1**

A list of microRNAs with respective top 15 and least 15 PCA scores (correlation value) for each microRNA for the component 2 (Y-axis), which separated MMD and control, based on the principal component analysis for the 309 plasma-microRNAs (Please see Figure 2b).

| **Top 15 plasma microRNAs negatively correlated for the Component 2** | | | |
| --- | --- | --- | --- |
|  |  |  |  |
| microRNA_name | | PCA score for component 2 | |
|  |  |  |  |
| hsa-miR-4665-3p | | -3.70769 | |
| hsa-miR-6722-3p | | -3.42404 | |
| hsa-miR-4767 | | -3.32595 | |
| hsa-miR-6800-5p | | -3.25527 | |
| hsa-miR-762 | | -3.19543 | |
| hsa-miR-1234-3p | | -3.12233 | |
| hsa-miR-6089 | | -3.08291 | |
| hsa-miR-6797-3p | | -3.07845 | |
| hsa-miR-4532 | | -2.99268 | |
| hsa-miR-3610 | | -2.94765 | |
| hsa-miR-328 | | -2.92068 | |
| hsa-miR-6777-3p | | -2.90789 | |
| hsa-miR-6850-5p | | -2.87667 | |
| hsa-miR-150 | | -2.87138 | |
| hsa-miR-3162-3p | | -2.81739 | |
|  | |  | |
| **Top 15 plasma microRNAs positively correlated for the Component 2** | | |  |
|  |  |  |  |
| microRNA_name | PCA score for component 2 | |  |
|  |  |  |  |
| hsa-miR-4481 | 3.60522 | |  |
| hsa-miR-181b-5p | 3.49547 | |  |
| hsa-miR-595 | 3.42647 | |  |
| hsa-miR-8071 | 3.40804 | |  |
| hsa-miR-181d-5p | 3.34942 | |  |
| hsa-miR-1224-5p | 3.32213 | |  |
| hsa-miR-7845-5p | 3.28521 | |  |
| hsa-miR-6840-3p | 3.22997 | |  |
| hsa-miR-188-5p | 3.18272 | |  |
| hsa-miR-4701-3p | 3.12889 | |  |
| hsa-miR-4758-5p | 3.02474 | |  |
| hsa-miR-5196-5p | 2.98088 | |  |
| hsa-miR-6860 | 2.95921 | |  |
| hsa-miR-623 | 2.75108 | |  |
| hsa-miR-6756-5p | 2.74346 | |  |
