# Supplementary material for: Circulating miRNome profiling in Moyamoya disease-discordant monozygotic twins and endothelial microRNA expression analysis using iPS cell line
Source: BMC Med Genomics. 2018 Aug 29;11:72. doi: 10.1186/s12920-018-0385-3 (PMC6114494; doi:10.1186/s12920-018-0385-3)
Supplement: Supplementary file 3 — Table S2. Number of filtered target genes and target gene symbols for each of 17 differential circulating microRNAs in MMD, predicted by microRNA Target Filter implemented in IPA® (QIAGEN) and the miRmap web interface. The target genes were filtered based on their biological function analyzed using “Bioprofiler” implemented in IPA® (QIAGEN). As there is some overlap across microRNAs with respect to their target genes, so the total number of target genes at the bottom of the list is not a simple summation of each number of target genes. (DOCX 17 kb) [file 12920_2018_385_MOESM3_ESM.docx]

| **Table S2** |  |  |
| --- | --- | --- |
| **Number of filtered target genes and target gene symbols for each of 17 differential circulating microRNAs in MMD, predicted by microRNA Target Filter implemented in IPA^®^ (QIAGEN) and the miRmap web interface** | | |
| The target genes were filtered based on their biological function analyzed using “Bioprofiler” implemented in IPA^®^ (QIAGEN). As there is some overlap across microRNAs with respect to their target genes, so the total number of target genes at the bottom of the list is not a simple summation of each number of target genes. | | |
|  |  |  |
| microRNA Symbol (mature microRNA) | microRNA ID | Number of targeted genes (filtered) |
| miR-1909-3p (and other miRNAs w/seed GCAGGGG) | hsa-miR-6722-3p |  |
|  | hsa-miR-6850-5p | 287 |
| miR-762 (and other miRNAs w/seed GGGCUGG) | hsa-miR-762 | 329 |
| miR4532 (miRNAs w/seed CCCGGGG) | hsa-miR-4532 | 79 |
| miR-6089 (miRNAs w/seed GAGGCCG) | hsa-miR-6089 | 176 |
| miR-328-3p (and other miRNAs w/seed UGGCCCU) | hsa-miR-328 | 48 |
| miR-328-5p (and other miRNAs w/seed GGGGGGC) |  | 211 |
| miR-6808-5p (and other miRNAs w/seed AGGCAGG) | hsa-miR-940 | 32 |
| miR-6800-5p (miRNAs w/seed UAGGUGA) | hsa-miR-6800-5p | 8 |
| miR-718 (miRNAs w/seed UUCCGCC) | hsa-miR-718 | 15 |
| miR-150-3p (miRNAs w/seed UGGUACA) | hsa-miR-150 | 48 |
| miR-150-5p (and other miRNAs w/seed CUCCCAA) |  | 102 |
| miR-3665 (miRNAs w/seed GCAGGUG) | hsa-miR-3665 | 85 |
| miR-3610 (miRNAs w/seed AAUCGGA) | hsa-miR-3610 | 3 |
| miR-5008-3p (and other miRNAs w/seed CUGUGCU) | hsa-miR-6737-3p | 110 |
| miR-623 (miRNAs w/seed UCCCUUG) | hsa-miR-623 | 68 |
| miR-595 (miRNAs w/seed AAGUGUG) | hsa-miR-595 | 35 |
| miR-7845-5p (miRNAs w/seed AGGGACA) | hsa-miR-7845-5p | 118 |
| miR-4481 (and other miRNAs w/seed GAGUGGG) | hsa-miR-4481 | 98 |
| Number of genes (union) | | 1069 |
