# Supplementary figures and images for: Circulating miRNome profiling in Moyamoya disease-discordant monozygotic twins and endothelial microRNA expression analysis using iPS cell line
Source: BMC Med Genomics. 2018 Aug 29;11:72. doi: 10.1186/s12920-018-0385-3 (PMC6114494; doi:10.1186/s12920-018-0385-3)

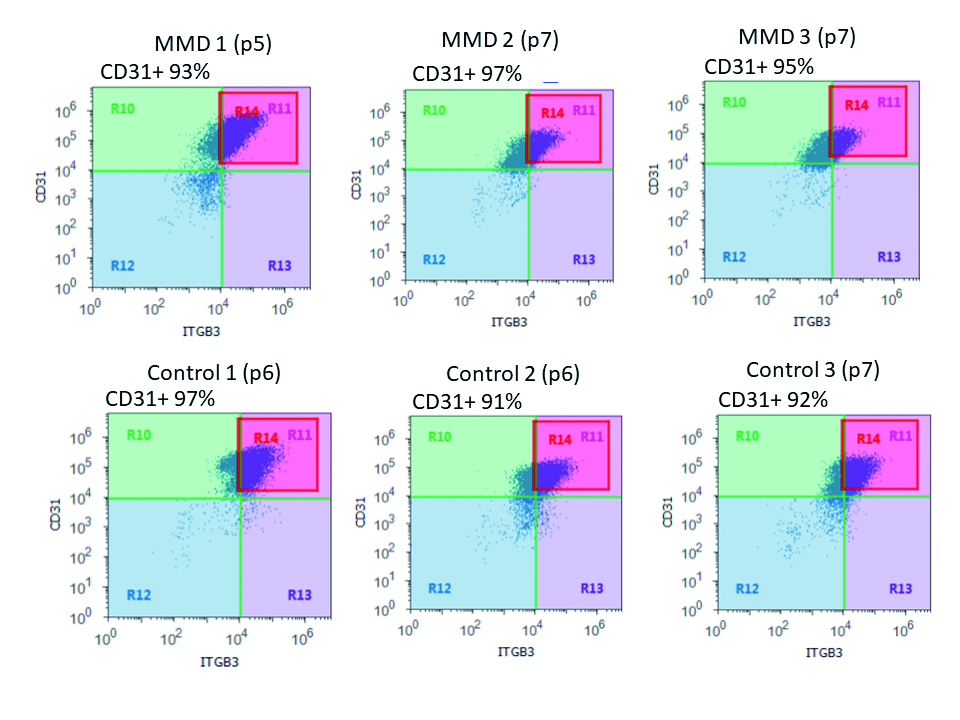

Supplement: Supplementary file 4 — Figure S1. FACS plot to confirm the purity of iPSECs. The purity of the iPSECs after 5–6 times passages was confirmed as high as 91–97% in all clones using anti-CD31 antibody with the FACS. (TIF 1011 kb) [file 12920_2018_385_MOESM4_ESM.tif]
