# Supplementary material for: Circulating miRNome profiling in Moyamoya disease-discordant monozygotic twins and endothelial microRNA expression analysis using iPS cell line
Source: BMC Med Genomics. 2018 Aug 29;11:72. doi: 10.1186/s12920-018-0385-3 (PMC6114494; doi:10.1186/s12920-018-0385-3)
Supplement: Supplementary file 5 — Table S3. The 41 target genes of hsa-miR-6722-3p/328-3p that were significantly down-regulated in MMD-iPSECs. Expression log2 fold change (log2 FC), p-value, and q-value were obtained from our previous publication (Hamauchi et al. 2016 [12]). Molecule type for each gene was obtained from the IPA tool. (DOCX 19 kb) [file 12920_2018_385_MOESM5_ESM.docx]

| **Table S3** | |  |  |  |  |
| --- | --- | --- | --- | --- | --- |
| **The 41 target genes of hsa-miR-6722-3p/328-3p that were significantly down-regulated in MMD-iPSECs** | | | | | |
| Expression log2 fold change (log2 FC), p-value, and q-value were obtained from our previous publication (Hamauchi et al. 2016 [12]). Molecule type for each gene was obtained from the IPA tool. | | | | | |
|  |  |  |  |  |  |
| **Gene Symbol** | **Gene Name** | **log2FC** | **q-value** | **p-value** | **Molecule Type** |
| **ADAM12** | ADAM metallopeptidase domain 12 | -2.04 | **0.0825** | **0.0013** | peptidase |
| **AGRN** | agrin | -1.03 | **0.0821** | **0.0010** | other |
| **AHDC1** | AT-hook DNA binding motif containing 1 | -0.61 | **0.1122** | **0.0117** | other |
| **ASXL2** | additional sex combs like 2, transcriptional regulator | -0.65 | **0.1336** | **0.0225** | other |
| **ASXL3** | additional sex combs like 3, transcriptional regulator | -1.89 | **0.0861** | **0.0020** | other |
| **ATP7A** | ATPase copper transporting alpha | -0.72 | **0.1431** | **0.0292** | transporter |
| **ATP8A2** | ATPase phospholipid transporting 8A2 | -1.03 | **0.1468** | **0.0319** | transporter |
| **BICD2** | BICD cargo adaptor 2 | -1.22 | **0.0821** | **0.0006** | other |
| **BMPR2** | bone morphogenetic protein receptor type 2 | -1.06 | **0.1404** | **0.0276** | kinase |
| **CDH5** | cadherin 5 | -0.63 | **0.1195** | **0.0153** | other |
| **CDK6** | cyclin dependent kinase 6 | -1.98 | **0.0962** | **0.0051** | kinase |
| **CLCN4** | chloride voltage-gated channel 4 | -1.38 | **0.1034** | **0.0083** | ion channel |
| **CLN8** | CLN8, transmembrane ER and ERGIC protein | -1.03 | **0.1004** | **0.0066** | other |
| **CNNM2** | cyclin and CBS domain divalent metal cation transport mediator 2 | -0.87 | **0.0946** | **0.0041** | transporter |
| **COL4A2** | collagen type IV alpha 2 chain | -0.98 | **0.1146** | **0.0125** | other |
| **CTSA** | cathepsin A | -0.62 | **0.1157** | **0.0134** | peptidase |
| **CTSB** | cathepsin B | -0.69 | **0.1071** | **0.0096** | peptidase |
| **DCHS1** | dachsous cadherin-related 1 | -1.01 | **0.1451** | **0.0308** | other |
| **EPG5** | ectopic P-granules autophagy protein 5 homolog | -0.99 | **0.0894** | **0.0029** | other |
| **FBXW7** | F-box and WD repeat domain containing 7 | -0.66 | **0.1137** | **0.0122** | transcription regulator |
| **FGF2** | fibroblast growth factor 2 | -1.00 | **0.0920** | **0.0035** | growth factor |
| **FOXO3** | forkhead box O3 | -1.52 | **0.1117** | **0.0114** | transcription regulator |
| **IGF1R** | insulin like growth factor 1 receptor | -1.07 | **0.1212** | **0.0164** | transmembrane receptor |
| **MBOAT7** | membrane bound O-acyltransferase domain containing 7 | -0.60 | **0.1400** | **0.0271** | enzyme |
| **MCAM** | melanoma cell adhesion molecule | -0.66 | **0.1390** | **0.0265** | other |
| **PACS1** | phosphofurin acidic cluster sorting protein 1 | -1.04 | **0.1442** | **0.0299** | other |
| **PAPPA** | pappalysin 1 | -1.84 | **0.1205** | **0.0161** | peptidase |
| **PGAP1** | post-GPI attachment to proteins 1 | -1.15 | **0.1286** | **0.0200** | enzyme |
| **PTPRJ** | protein tyrosine phosphatase, receptor type J | -1.58 | **0.0831** | **0.0015** | phosphatase |
| **RAB18** | RAB18, member RAS oncogene family | -1.13 | **0.1385** | **0.0262** | enzyme |
| **RNF213** | ring finger protein 213 | -1.63 | **0.0969** | **0.0056** | enzyme |
| **SH3PXD2A** | SH3 and PX domains 2A | -1.48 | **0.0821** | **0.0001** | other |
| **SH3PXD2B** | SH3 and PX domains 2B | -0.67 | **0.1341** | **0.0229** | other |
| **SLC30A7** | solute carrier family 30 member 7 | -0.64 | **0.1244** | **0.0180** | transporter |
| **SLC6A8** | solute carrier family 6 member 8 | -1.04 | **0.1082** | **0.0102** | transporter |
| **SON** | SON DNA binding protein | -0.78 | **0.0986** | **0.0061** | other |
| **STAT3** | signal transducer and activator of transcription 3 | -0.77 | **0.1004** | **0.0068** | transcription regulator |
| **TPP1** | tripeptidyl peptidase 1 | -1.54 | **0.0821** | **0.0004** | peptidase |
| **VAPB** | VAMP associated protein B and C | -1.03 | **0.0941** | **0.0040** | other |
| **VPS13A** | vacuolar protein sorting 13 homolog A | -0.69 | **0.1157** | **0.0135** | transporter |
| **WNK1** | WNK lysine deficient protein kinase 1 | -0.82 | **0.1004** | **0.0067** | kinase |
